# Passive Type Reconfigurable Intelligent Surface: Measurement of Radiation Patterns

**DOI:** 10.3390/mi14040818

**Published:** 2023-04-05

**Authors:** Biswarup Rana, Sung-Sil Cho, Ic-Pyo Hong

**Affiliations:** 1 Smart Natural Space Research Centre, Kongju National University, Cheonan 31080, Republic of Korea; 2 Department of Smart Information Technology Engineering, Kongju National University, Cheonan 31080, Republic of Korea

**Keywords:** reconfigurable intelligent surface, beyond-5G/6G, measurement, unit cell, passive

## Abstract

The demand for unprecedented data and ubiquitous wireless connections have led to the adoption of new types of transmitters and receivers. Additionally, different new types of devices and technologies need to be proposed for such demand. Reconfigurable intelligent surface (RIS) is going to play a very significant role in the upcoming beyond-5G/6G communications. It is envisioned that not only the RIS will be deployed to assist and create a smart wireless environment for the upcoming communications, but also the receiver and transmitter can be fabricated using RIS to make a smart and intelligent transmitter and receiver. Thus, the latency of upcoming communications can be reduced very significantly using RIS, which is a very important factor. Artificial intelligence assists communications and shall be adopted widely for the next generation networks. In this paper, radiation pattern measurement results of our previously published RIS have been provided. This work is the extension work of our previously proposed RIS. The polarization-independent passive type of RIS working in the sub-6 GHz frequency band using low-cost FR4-substrate was designed. Each unit cell with dimensions of 42 mm × 42 mm had a single-layer substrate backed by a copper plate. A 10 × 10-unit cell array was fabricated to check the performance of the RIS. Such types of unit cells and RIS were designed to set up initial measurement facilities in our laboratory for any kinds of RIS measurements.

## 1. Introduction

RIS is emerging as one of the main technologies for beyond-5G/6G communications. By changing the properties of the surface of the RIS, a programmable structure can be conceived to control the electromagnetic waves [1]. This programable structure can be placed in the desired locations to improve the performance of wireless communications. Although 5G is now in the initial stage, researchers and engineers across the world are looking forward to the technology and applications for 6G communications [2,3,4,5,6]. Massive multiple-input multiple-output (MIMO) is one of the main technologies for the current 5G communications [7,8,9,10,11]. A large number of antennas, typically 64 or more antenna arrays, are being used for 5G communications. The 6G will have some applications that cannot be fully obtained using the current 5G technology. These services and applications are augmented reality [12], virtual reality, mixed reality, brain–machine interface [13], indoor localization [14], connected health [15], connected unmanned aerial vehicles [16], smart cities [17], connected robotics, connected autonomous vehicles [18], etc. The 6G communications will have some features such as (a) higher spectral and energy efficiency; (b) less than 1 ms latency [19]; (c) 1Tb/s data rate [19]; (d) very wide frequency bands such as sub-6 GHz, mmWave frequency bands, THz bands, optical frequency bands etc. [19]; (e) 99.99999% end to end reliability [19]; (f) intelligent transmitter and receiver with RIS to create a smart wireless environment, etc. It is expected that RIS will be deployed to make smart wireless environments beyond 5G/6G communications [20,21,22,23,24,25,26,27,28,29,30]. Recently, there has been very significant research activity in this area. Different researchers and engineers are focusing on different areas of the RIS such as theoretical approaches, channel modeling, communication performance improvement, design and fabrication of the RIS, etc. The spectral and energy efficiency of a network can be improved significantly using RIS. RIS also supports full duplex communications. Normally RIS are constructed by a metasurface, with subwavelength dimensions of each unit cell. Nowadays, most authors are focusing on reflective types of RIS. However, the present authors envision that transmissive types of RIS will be also useful for beyond-5G/6G communications. The amplitude and phase of each unit cell of the RIS can be tuned to obtain the reconfigurable features. Normally, microcontrollers or field programmable gate arrays (FPGAs) are used as a controller of the RIS. However, other control mechanisms are also being implemented.

In the literature, a significant number of RIS hardware have been proposed. Most of the authors used PIN diodes or varactor didoes to design the RIS in the sub-6 GHz frequency band or mmWave frequency band. However, most of the published papers in the literature are very complex, and those structures need a complex controller to change the phase or amplitude of the reflected waves. In [31,32], the authors proposed an all-metal wideband phase correcting structure and an all-metal wideband frequency-selective surface bandpass filter, respectively. These types of all-metal structures can be useful to design RIS. Recently, artificial intelligence has been brought to the design of intelligent surfaces, where different nature-based algorithms have been used to design such surfaces [33]. In the literature, there are a very large number of papers available on RIS, regarding different aspects. Nowadays, PIN and varactor diodes are the most popular tunning devices to design RIS. In [34,35], the authors used varactor diodes to design RIS at the sub-6 GHz frequency band. PIN diodes were used to design RIS in [25,36]. Not only reflective types of RIS but also transmissive types of RIS will be used for next-generation communications [37]. Graphene is a material that will be useful to design RIS [38,39]. It is expected that multi-functional RISs are going to deploy beyond-5G/6G communications [40,41]. The multifunctional RISs shall have extra capabilities such as sensing the incoming signal. VO_2_ and liquid crystals are other types of promising tuning materials that can be used to design RIS. In [42], the authors used VO_2_ to design RIS by using the metal-to-insulator property of VO_2_. Liquid crystal-based RIS having multi-resonant cells was proposed in [43]. In [44], the present authors proposed a passive type of polarization-independent RIS at the sub-6 GHz frequency band. Any types of polarization-related losses could be overcome using that structure. The proposed RIS was fabricated on a low-cost FR4 substrate backed by a copper plate. The reconfigurability of that proposed RIS could be achieved by simply rotating the entire RIS. We designed this type of RIS because, initially, we wanted to establish a measurement facility in our laboratory for any kind of RIS measurement. It is a relatively complex task to obtain the radiation patterns for the RIS, as the transmitting and receiving antennas are far from the RIS. However, we are able to obtain simulated and measured radiation patterns for our proposed RIS. The main novelty of our work is that in our paper we have described a measurement technique of the scanned beam of a passive type of RIS. Our measurement process described in this paper will be useful for the quick initial performance evaluation of any type of RIS before actual deployment. In this paper, we have provided the measurement results of the reflected radiation pattern of our previously proposed RIS [44].

## 2. Configuration of Unit Cells and 10 × 10-Unit Cell Arrays

**Figure 1:**
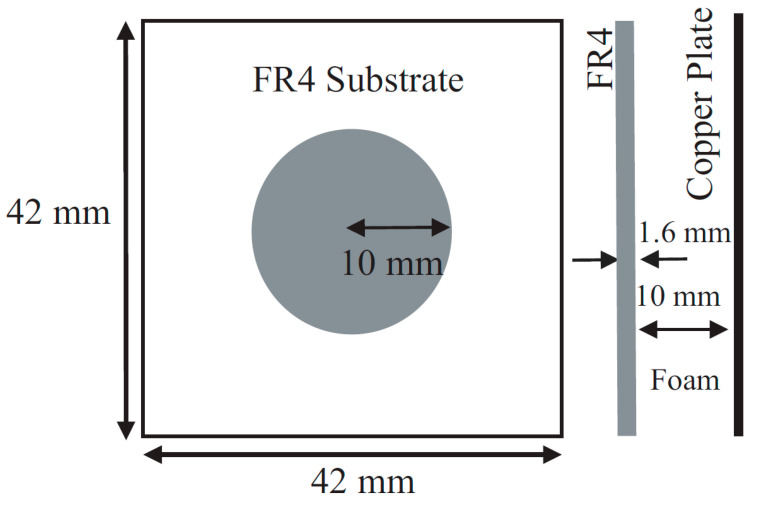
Top view and side view of the circular patch-type unit cell.

**Figure 2:**
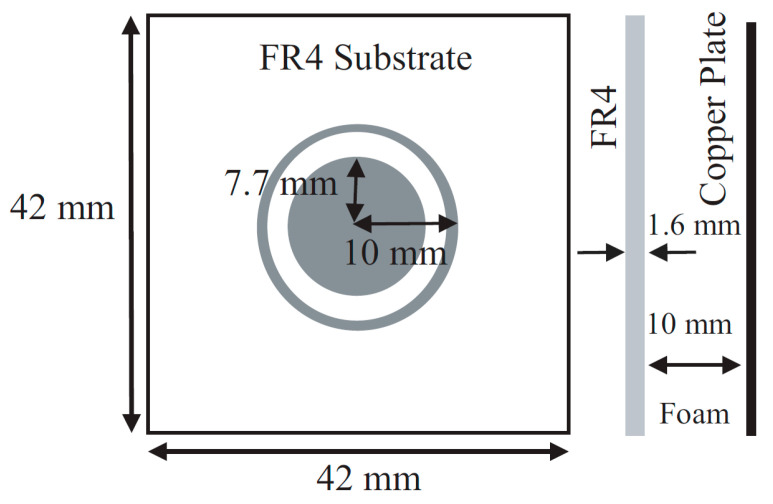
Top view and side view of the circular patch with a ring-type unit cell.

**Figure 3:**
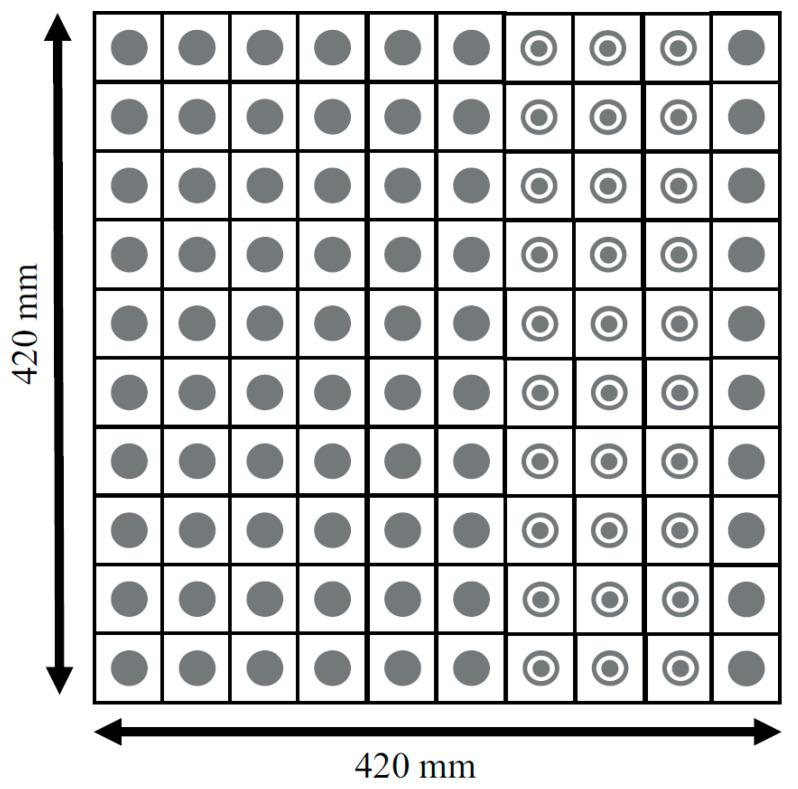
Configuration of the 10 × 10 RIS.

**Figure 4:**
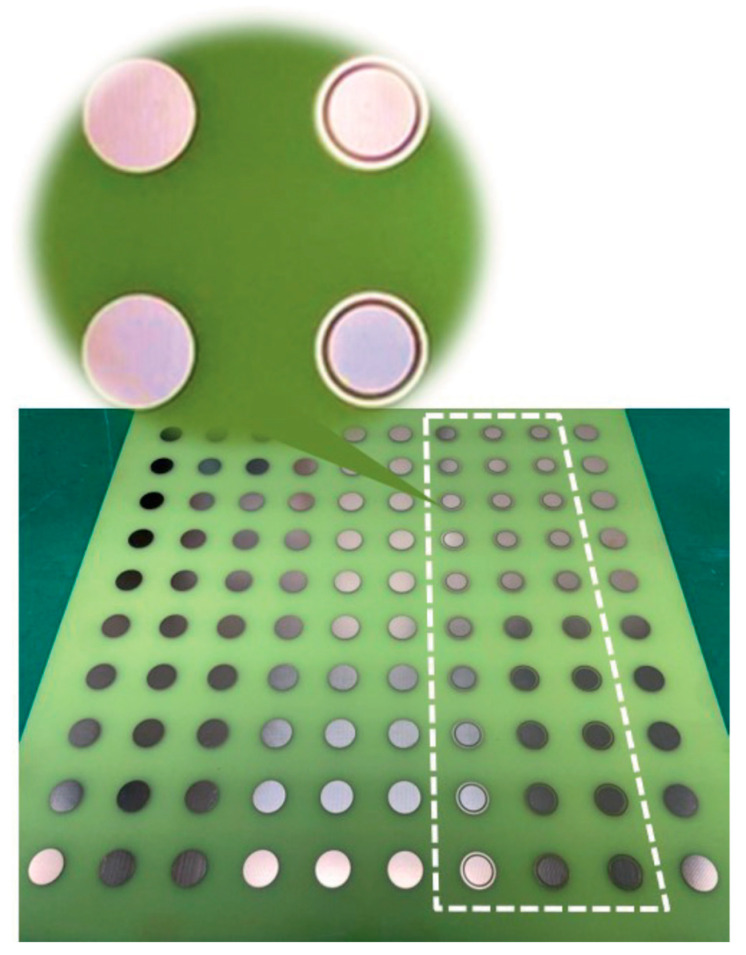
Fabricated 10 × 10 RIS.

Metamaterials that show unique properties are not available in nature. These engineered materials need to be designed and fabricated artificially. Two-dimensional versions of the metamaterial are the metasurface. There are a wide variety of applications of the RIS such as cloaking, flat lenses, hologram, optical encryption, polarizers, modulators, noise cancellation, ultrasound imaging, etc. RIS can be fabricated using (a) reconfigurable metasurface and (b) reconfigurable patch type reflectarray or transmitarray antenna. In the case of metasurface-based RIS, the distance between unit cells is sub-wavelength, normally λ_0_/10 or below. However, in the case of reconfigurable reflectarray or transmit array-based RIS, the distance between unit cells is normally set to λ_0_/2. However, the distance between the unit cells can be lower or greater than λ_0_/2 for the reconfigurable patch reflectarray or transmitarray antenna. Simple and cost-effective are the two features that are essential to deploy the RISs in real scenarios. In [44], the details of our proposed unit cell configurations and three types of 10 × 10 arrays were discussed. The proposed unit cells were very cost-effective, and they were very easy to design and fabricate. Expensive substrates can increase the overall cost to deploy the RIS. We have used an FR4 substrate to check the initial performance of the RIS, and the proposed configuration can be designed on a low-loss substrate also with improved performance. Initially, a circular type of patch unit cell was designed. Then, a circular type of patch with a ring unit cell was designed. Lastly, three 10 × 10 unit cell arrays with the proposed unit cells were designed and fabricated. We have taken the circular type of structure because the structure is symmetric. So, the structure can reflect any type of incident polarization. The structure is a polarization-independent structure. Thus, polarization-related losses can be overcome using this structure. The design frequency is 3.5 GHz. The free space half wavelength at 3.5 GHz is 42.86 mm. The proposed unit cell size is 42 mm × 42 mm which is almost λ_0_/2 mm × λ_0_/2 mm at 3.5 GHz. With such space between the unit cell, it is possible to reduce the mutual coupling between the unit cells. Additionally, with such space, it is possible to get rid of the grating lobes of the reflected radiation pattern. The cell alignment is taken from the conventional phased array antenna theory. With such phase alignment, we can obtain a steered radiation pattern. Ansys Electronics Desktop was used to simulate the unit cells, arrays, and measurement environment. While simulating the unit cell, we used Floquet port master slave boundary conditions to obtain the simulated magnitude and phase of the reflection coefficients. We used a 10 × 10 array in absorbing boundary conditions to obtain reflected radiation patterns. Around the 10 × 10-unit cells, we have put a perfectly matched layer (PML) boundary so that the impinging wave on the RIS can get reflected back properly without any type of reflection from the other place except 10 × 10 RIS. Figure 1 shows the top view and side view of the proposed circular type of unit cell, while Figure 2 shows the top view and side view of the proposed circular type of patch with a ring unit cell. A low-cost FR4 substrate with a thickness of 1.6 mm, permittivity of 4.4, and a loss tangent of 0.02 was considered while designing the unit cells and arrays. The target frequency was 3.5 GHz, and the area of the unit cell was 42 mm × 42 mm. A foam and copper plate as shown in Figure 1 and Figure 2 was used. The circular-shaped structure enabled the polarization independence nature of the unit cell because the unit cell was symmetric in a plane. The proposed 10 × 10 RIS was designed using two types of unit cells, as shown in Figure 3. The fabricated prototype is shown in Figure 4.

## 3. Measurement of Radiation Patterns of the RISs

**Figure 5:**
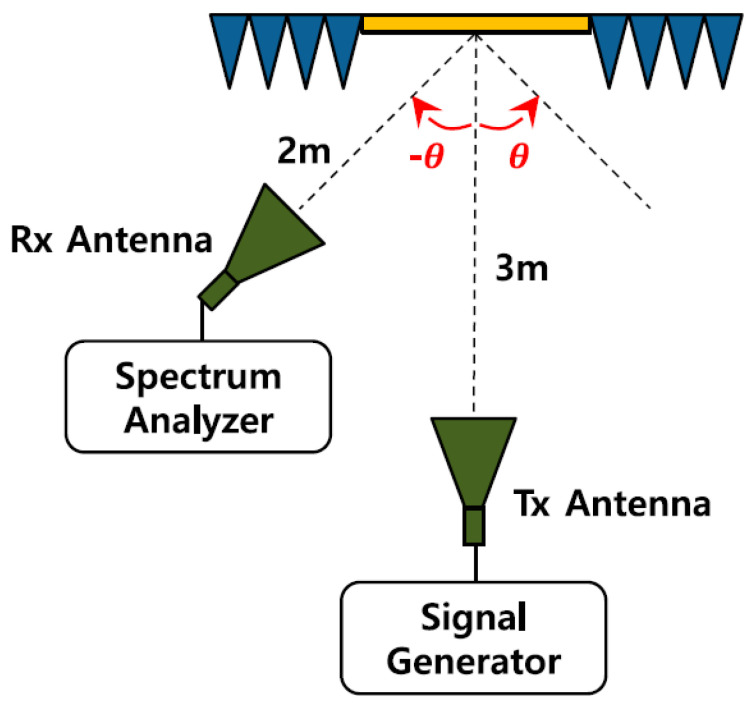
Schematic diagram of the radiation patterns measurement setup for the RIS.

The actual picture of the measurement setup was presented in [44]. In this paper, we have focused on and presented the radiation pattern measurement. The measurement setup is as follows:

(a) The measurement setup was as shown in Figure 5.
(b) One absorbing board was in front of the horn antenna.
(c) Middle portion of the absorbing board, the absorbing material was removed, and the proposed RIS was placed.
(d) Two more absorbing boards were placed to minimize the reflections from the sounding walls and to remove the unwanted signals from the outside.

The measurement procedure is as follows:

(a) As shown in Figure 5, the signal generator was connected to the transmitting horn antenna, and the spectrum analyzer was connected to the receiving horn antenna.
(b) The fixed transmitting horn antenna was at a distance of 3 m from the RIS.
(c) The receiving horn antenna was placed at a distance of 2 m from the RIS.
(d) The receiving horn antenna was rotated up to an angle of 20° and −20° with a step of 5°.
(e) In order to check how much signal was reflected compared to the same area at the measurement point, a reference value was first measured using a reflector, and then the received signal strength of the passive RIS was measured.

**Figure 6:**
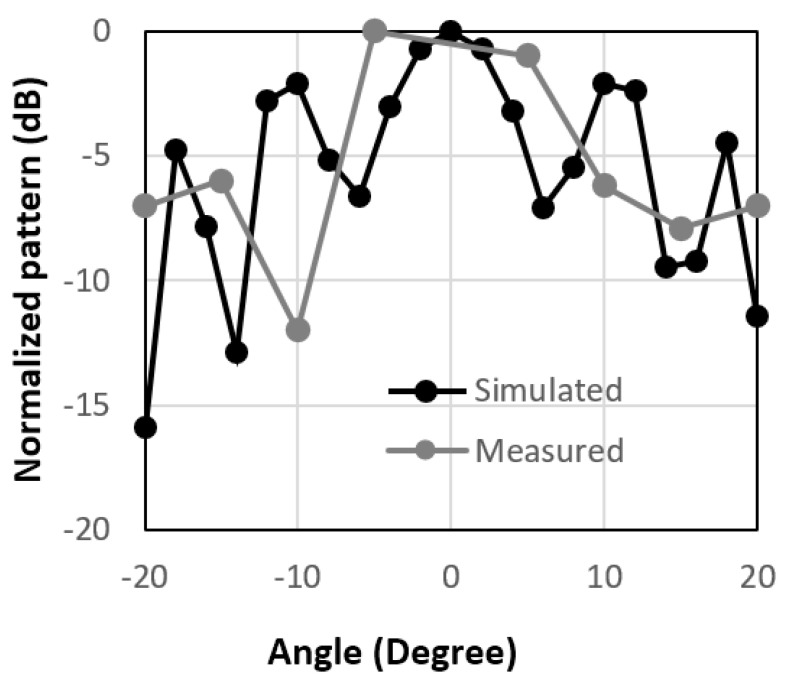
Normalized radiation patterns for circular patch-type unit cells at 3.5 GHz.

**Figure 7:**
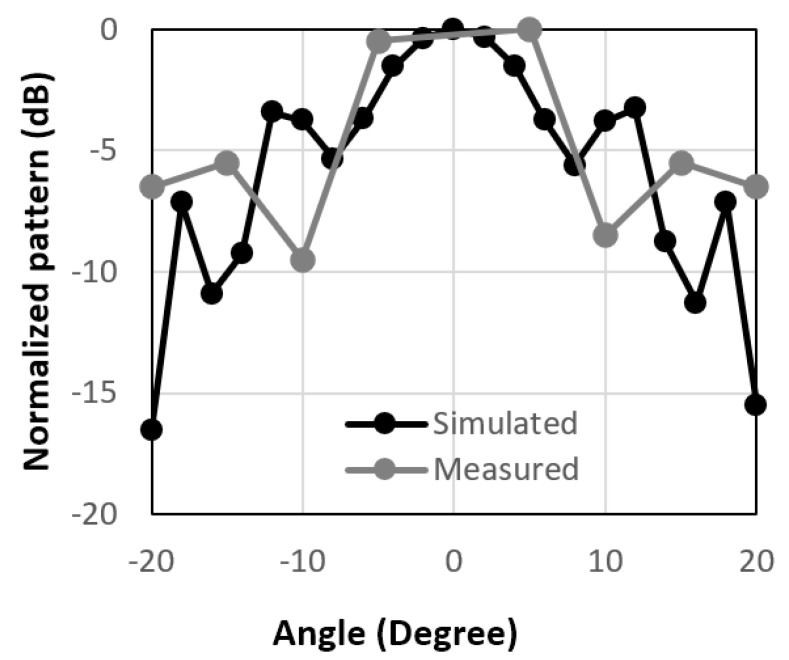
Normalized radiation patterns for circular patch with ring type of unit cells at 3.5 GHz.

**Figure 8:**
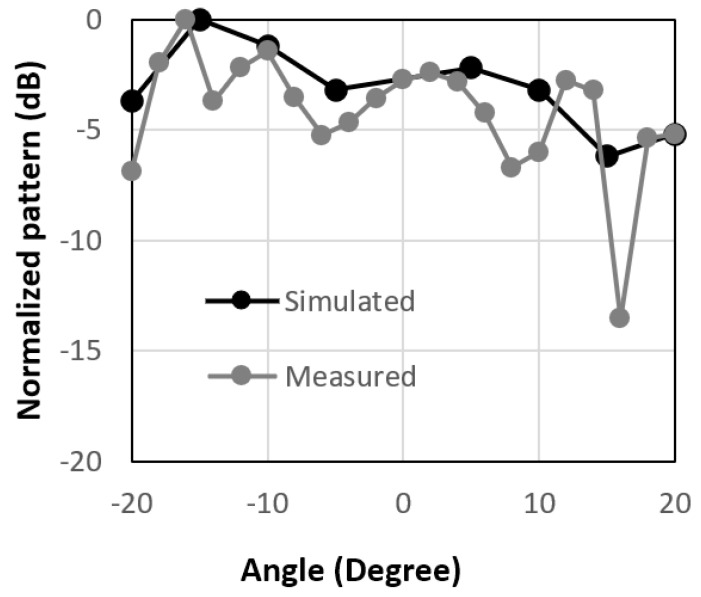
Normalized radiation patterns for combined-type of unit cells at 3.5 GHz.

Figure 6, Figure 7 and Figure 8 show the radiation patterns for different 10 × 10-unit cell arrays, namely circular type patch, circular type patch with a ring, and the combined structure. The 10 × 10 circular type patch unit cell array had the same type of unit cells. So, the incident angle and reflected angle are the same for this type of array and the maximum received power would have been at 0°. However, due to the large size of the receiving and transmitting horn antennas, it was not possible to measure the received power at 0°.

Overall, the peak measured value of the received power for the 10 × 10-unit cell arrays was not at 0°, as shown in Figure 6. The same thing is also valid for the 10 × 10 circular type patch with a ring unit cell array, as shown in Figure 7. To reflect the beam in a different direction, a 10 × 10 combined structure was fabricated and the radiation performance of the reflected is shown in Figure 8. It is observed from the figure that the measured reflected beam from the combined RIS was at about 15° for the incident angle of 0°, while the simulated peak value was at 16°. So, from this result, an anomalous/engineered reflection was observed. There was some mismatch between the simulated and measured results because of the scattering from the edge of the RIS and the fabrication inaccuracy of the RIS. The results and design are very promising because a single-layer low-cost FR4 substrate backed by a copper plate was used to design the proposed unit cell array. In some applications, it is necessary to use low-cost RIS with a much less complex controller. The whole RIS can be rotated mechanically at 0°, 90°, 180°, and 270° to obtain engineered reflected radiation patterns. It is expected that these passive types of this RIS will be useful where it is necessary to change the radiation patterns a few times daily. The proposed passive RIS also will be useful to cover the blind spots where there is no need to change the direction of the radiation pattern dynamically.

## 4. Conclusions

In this paper, the radiation patterns of our previously proposed passive types of RIS have been verified. The work is an extension of our previously published paper. A RIS working at the sub-6 GHz frequency band was designed. We designed three types of 10 × 10 RIS: circular type patch, circular type patch with a ring, and combined structure. We checked radiation patterns for each 10 × 10-unit cell array. For a 10 × 10-unit cell array with a circular patch, and a 10 × 10-unit cell array with a circular patch with a ring, the radiation patterns were normal radiation patterns. However, for the combined types of RIS, the peak of received signal power was at 15°. Rotating the combined structure using some mechanical motor, pattern reconfigurability can be achieved. The measurement process of the proposed RIS is promising, and the initial performance evaluation of any kind of RIS can be obtained using the process described in this paper.
